# Integrated mental health care and vocational rehabilitation to improve return to work rates for people on sick leave because of depression and anxiety (the Danish IBBIS trial): study protocol for a randomized controlled trial

**DOI:** 10.1186/s13063-017-2272-1

**Published:** 2017-12-02

**Authors:** Rie Poulsen, Andreas Hoff, Jonas Fisker, Carsten Hjorthøj, Lene Falgaard Eplov

**Affiliations:** Mental Health Center Copenhagen, Mental Health Services Capital Region of Denmark, University of Copenhagen, Kildegårdsvej 28, Opgang 15.4, DK-2900 Hellerup, Copenhagen Denmark

**Keywords:** Depression, Anxiety, Return to work, Integrated services, Mental health care, Cognitive behavioral therapy, Vocational rehabilitation, Prevention of recurrent sickness absence, RCT, Common mental disorders

## Abstract

**Background:**

Depression and anxiety are among the largest contributors to the global burden of disease and have negative effects on both the individual and society. Depression and anxiety are very likely to influence the individual’s work ability, and up to 40% of the people on sick leave in Denmark have depression and/or anxiety. There is no clear evidence that treatment alone will provide sufficient support for vocational recovery in this group. Integrated vocational and health care services have shown good effects on return to work in other, similar welfare contexts. The purpose of the IBBIS (Integrated Mental Health Care and Vocational Rehabilitation to Individuals on Sick Leave Due to Anxiety and Depression) interventions is to improve and hasten the process of return to employment for people in Denmark on sick leave because of depression and anxiety.

**Methods/design:**

This three-arm, parallel-group, randomized superiority trial has been set up to investigate the effectiveness of the IBBIS mental health care intervention and the integrated IBBIS mental health care and IBBIS vocational rehabilitation intervention for people on sick leave because of depression and/or anxiety in Denmark. The trial has an investigator-initiated multicenter design. A total of 603 patients will be recruited from Danish job centers in 4 municipalities and randomly assigned to one of 3 groups: (1) IBBIS mental health care integrated with IBBIS vocational rehabilitation, (2) IBBIS mental health care and standard vocational rehabilitation, and (3) standard mental health care and standard vocational rehabilitation. The primary outcome is register-based return to work at 12 months. The secondary outcome measures are self-assessed level of depression (Beck Depression Inventory II), anxiety (Beck Anxiety Inventory), stress symptoms (Four-Dimensional Symptom Questionnaire), work and social functioning (Work and Social Adjustment Scale), and register-based recurrent sickness absence.

**Discussion:**

This study will provide new knowledge on vocational recovery, integrated vocational and health care interventions, and prevention of recurrent sickness absence among people with depression and anxiety. If the effect on return to work is different in the intervention groups, this study can contribute to current knowledge on shared care models for health care and vocational rehabilitation services.

**Trial registration:**

ClinicalTrials.gov, NCT02872051. Retrospectively registered on 15 August 2016.

**Electronic supplementary material:**

The online version of this article (doi:10.1186/s13063-017-2272-1) contains supplementary material, which is available to authorized users.

## Background

Depression and anxiety affect individuals’ psychosocial and occupational functioning [1–3] and are increasing causes of sick leave in many high-income countries [4, 5]. In Denmark, around 40% of all recipients of sick leave benefit have depression and/or anxiety [6], and neurotic diseases and depression are, respectively, the first and fourth most common reasons for early retirement on health grounds in Denmark [7]. Psychiatric illness has an estimated financial burden on the Danish economy at 3.4% of the Danish gross domestic product [5]. Common mental disorders such as anxiety and depression cause the largest financial burdens because of their high prevalence [5, 8].

Though work can impose difficult and stressful challenges for the individual, it also seems to be pivotal for people [9]. Manifest (e.g., income) and latent (e.g., daily structure, social contact, professional identity, status, and activity) benefits from employment make work attractive for the individual [10], similarly for people with mental disorders [11]. Furthermore, long-term sick leave and unemployment is a known stressor and a risk factor for poor mental health for the individual [12, 13]. Thus, it is highly relevant to help individuals on sick leave because of depression and/or anxiety to return to work and prevent further sickness absence and deterioration in mental health [14]. The aim of the IBBIS (Integrated Mental Health Care and Vocational Rehabilitation to Individuals on Sick Leave Due to Anxiety and Depression) project is to improve sick leave beneficiaries’ process of returning to employment after sick leave due to depression and anxiety.

Disease severity is an established predictor for return to work and work functioning for depressed people [15], and symptoms of anxiety (e.g., extensive worrying) are associated with poor employment outcomes [16]. Lowering the symptom level by providing better and more systematic detection and treatment options to people on sick leave because of depression and anxiety have been suggested to improve return to work [17, 18]. This might be feasible in Denmark, where detection and treatment of depression and anxiety in primary care is judged to be suboptimal and stepped care as it is described in Danish and international guidelines is not provided sufficiently [19–23]. Improved treatment alone has nonetheless not shown convincing results for improving return to work rates for people with depression [24], and symptom reduction is not the only important factor in the process of returning to work [25]. Several studies have investigated the effect of psychological interventions on return to work after depression, and the evidence is somewhat mixed [26–28]. A Cochrane review concluded that work-directed interventions should be provided in conjunction with treatment interventions to improve vocational recovery by addressing the barriers related to returning to work for people with depression [24]. Few studies have investigated interventions to improve return to work rates for employed people with anxiety disorders [29]. People with anxiety disorders are included in some studies, which have proven effective for earlier return to work for people with common mental disorders [30–32]. The substantial comorbidity between depression and anxiety justifies the assumption that people with common mental disorders need similar help to return to work. The subgroups of participants with anxiety disorders are very small, and return to work rates are generally not reported separately for anxiety disorders [30–33].

Return to work following sick leave is a multifaceted and complex process [34]. Personal, structural, and work-related factors probably also play an important role in the trajectory of return to work [25, 35]. Thus, the process of recovering from mental disorders and the process of vocational recovery are intertwined because the reintegration into the workplace affects the individual’s mental health just as well as mental health affects work reintegration [36].

The individual’s vocational recovery is influenced by factors other than the health care system: the employer, the colleagues, social security office, and vocational rehabilitation services. In Denmark, the latter two are governed by the same public office, called *job centers*. The Organisation for Economic Co-operation and Development suggests that there is an unfortunate lack of coordination between the health care system and social insurance offices in Scandinavian countries [5]. The lack of collaboration reduces the chances of early detection and referral of people with depression and anxiety to mental health care [17]. Most importantly, the lack of collaboration can cause conflicting requirements and goals and leave the individual on sick leave with a feeling of confusion and uncertainty at a time where the individual lacks control and certainty [34, 37].

Evidence on well-described integrated mental health and vocational rehabilitation models for people with common mental disorders is scarce. The Individual Placement and Support (IPS) intervention is an American intervention with a strong emphasis on integration of treatment and vocational support. IPS has been shown to be superior to standard services for people with severe mental illness attaining and maintaining work in several welfare countries [38, 39], but there is not yet solid evidence on how IPS can best be modified to suit a target group with common mental disorders. Recently, a large Norwegian study tested integrated employment support designed with an emphasis on IPS principles and work-directed therapy (At Work and Coping [AWaC]). The study showed positive results regarding faster return to work for people with common mental disorders [33]. Whereas IPS is normally provided within the health care system, services in the AWaC intervention are provided through the social security system. Intervention models that genuinely integrate services from the health care sectors and the employment sector in welfare countries have not yet, to our knowledge, been tested on a population with common mental disorders. The aim of the IBBIS trial for depression and anxiety is to test the effect on return to work from stepped mental health care and integrated stepped mental health care and vocational rehabilitation.

## Methods/design

### Aim

The aim of this randomized, three-arm, investigator-initiated, multicenter, parallel-group superiority trial is to compare the effect on return to work of the following interventions: (1) IBBIS mental health care integrated with IBBIS vocational rehabilitation, (2) IBBIS mental health care and standard vocational rehabilitation, and (3) standard mental health care and standard vocational rehabilitation. The primary hypothesis is that participants allocated to the IBBIS mental health care integrated with IBBIS vocational rehabilitation will have significantly faster return to work rates than people who are allocated to standard mental health care together with standard vocational rehabilitation. The secondary hypothesis is that IBBIS mental health care together with standard vocational rehabilitation will have a lesser but significant effect on return to work compared with standard mental health care together with standard vocational rehabilitation. The superiority of the IBBIS mental health care integrated with IBBIS vocational rehabilitation will also be tested by comparison with the IBBIS mental health care together with standard vocational rehabilitation intervention. The IBBIS vocational rehabilitation intervention alone will not be tested in this trial. We hypothesize that the superiority of the IBBIS interventions will be applicable for secondary outcomes and exploratory measures at 6-, 12-, and 24-months follow-up so that (1) symptom level and presenteeism will be lower in participants allocated to IBBIS interventions; (2) improvement in self-efficacy, quality of life, and functioning will be better among participants allocated to IBBIS interventions; and (3) satisfaction with services and number of weeks worked will be higher for participants allocated to IBBIS interventions.

The IBBIS trial is designed and reported in this article according to the Standard Protocol Items: Recommendations for Interventional Trials (SPIRIT) 2013 statement (see Additional file 1) [40], and the final results will be published according to the Consolidated Standards of Reporting Trials (CONSORT) criteria for randomized trials of nonpharmacological treatment [41]. There is a parallel trial in the IBBIS project with an identical research design for participants with adjustment disorder, exhaustion disorder, or psychological stress.

### Setting

The interventions will be delivered by a cross-sector and multidisciplinary IBBIS team that is organized in collaboration between Mental Health Services in the Capital Region of Denmark and the following four municipalities: the City of Copenhagen and the three suburban municipalities Gentofte, Gladsaxe, and Lyngby-Taarbæk. Participants are referred to the study by social security officers from job centers in the four municipalities, and the interventions are provided in locations other than the job centers and the mental health centers.

### Participants

Eligible participants in this trial meet the following inclusion criteria:

1. Are on sick leave from work or unemployment and have received sick leave benefit for at least 4 weeks or have started a sick leave benefit case that is estimated to last for at least 8 weeks
2. Meet the criteria for depression (F32.0-2 and F33.0-2), panic disorder (F41.0), social phobia (F40.1), or generalized anxiety disorder (F41.1) according to the Mini-International Neuropsychiatric Interview (M.I.N.I.) [42]
3. Are residents of collaborating municipalities at baseline
4. Are able to understand, speak, and read Danish
5. Are aged 18 years or older
6. Have given verbal and written consent to participate in the trial

Eligibility and diagnosis are assessed by an IBBIS team member (nurse, physiotherapist, social worker, occupational therapist, psychologist, or psychiatrist) who is trained to perform the assessment.

The IBBIS interventions are not designed to accommodate people in need of acute or highly specialized care. Thus, potential participants will not be eligible if they meet the exclusion criteria:

1. The assessor determines the patients’ suicide risk to be high according to the M.I.N.I. instrument [42], and the medical doctor confirms this risk.
2. The patient meets the screening criteria for dementia according to the Mini Mental State Examination (MMSE) screening instrument [43].
3. The patient abuses alcohol and/or other substances according to the assessor’s judgment.
4. The patient has a severe, unstable somatic condition (e.g., cancer, chronic obstructive pulmonary disease).
5. The patient needs secondary mental health care.
6. The patient is judged by job center staff to be at risk of displaying aggressive behaviors.

We wish to compare the IBBIS mental health care intervention alone with standard treatment. Thus, a seventh exclusion criterion is that a potential participant will not be included in the study if he or she does not accept the need to abstain from taking part in any psychotherapy or psychotherapy-like treatment outside the IBBIS intervention if he or she is randomized to IBBIS mental health care. Exclusion criteria 2–5 are applied only if the medical doctor on the IBBIS team confirms the assessment and anticipates that the patient cannot benefit from the IBBIS interventions. In cases with high suicide risk, the participant will be referred to acute care services. People with alcohol and substance abuse problems will be offered referral to treatment if relevant.

### Recruitment, data collection, and data management

Case managers from the four job centers can refer adult Danish-speaking citizens on sick leave from either work or unemployment to a psychiatric assessment if either the case manager, the citizen, or the individual’s general practitioner (GP) suspects a mental health condition to have caused the sick leave. The referral and assessment are voluntary. The results of the psychiatric assessment will be used in the treatment plan if the participant is allocated to treatment in IBBIS. The results of the assessment will be shared with the individual’s GP and the job center. The psychiatric assessment is based on three sources of information about the participant:

- Personal interview conducted by a care manager/psychologist and supervised by psychiatrist, guided by the following instruments:
  - M.I.N.I. [42]
  - Standardized Assessment of Personality–Abbreviated Scale [44]
  - Attention-deficit/hyperactivity disorder symptom checklist for adults (Adult ADHD Self-Report Scale version 1.1) [45]
  - If dementia is suspected, MMSE
- Self-assessed symptoms: Four-Dimensional Symptom Questionnaire (4DSQ) [46]
- GP’s sick leave note

Assessors are IBBIS team members who are specially trained to use the above-mentioned instruments. Trial eligibility will be evaluated after the psychiatric assessment, and subsequently, assessment data will constitute baseline data at the time −1 (*see* Table 1 and Additional file 1: Extended SPIRIT figure). The assessment process should not take more than 2 weeks but can be prolonged if one or more of the three types of information are missing.

**Table 1:** Standard Protocol Items: Recommendations for Interventional Trials (SPIRIT): enrollment and data collection

	Baseline (t_−1_)	Randomization (t_0_)	6-month follow-up (t_0_)	12-month follow-up (t_0_)	24-month follow-up (t_0_)
Informed consent	X				
CRF from personal interviews	X				
Randomization database		X			
Registration sheets	X	X	Continuous registration		
Self-assessment data	X		X	X	X
Register data		X	X	X	X

*CRF* Case report form

**Fig. 1:**
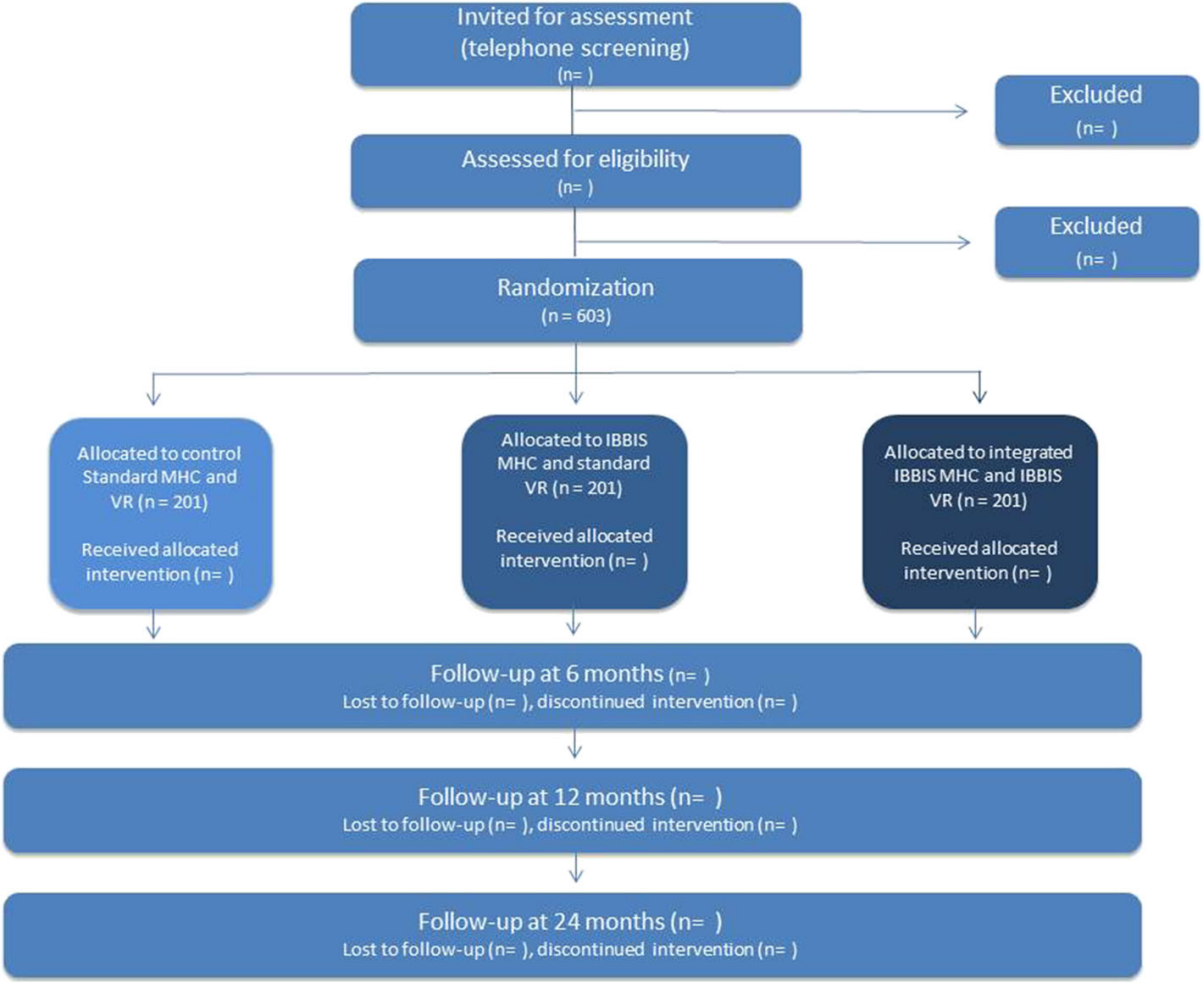
Flowchart of participant time line. *IBBIS* Integrated Mental Health Care and Vocational Rehabilitation to Individuals on Sick Leave Due to Anxiety and Depression, *MHC* Mental health center, *VR* Vocational rehabilitation

Individuals who meet the aforementioned trial criteria at assessment and subsequently consent to participate will be randomly allocated to an intervention by the assessor at t_0_. Participants will be followed at 6, 12, and 24 months after allocation (*see* Fig. 1). Participants will be prompted to fulfill self-assessment questionnaires at each follow-up through up to five personal contacts.

All electronic data (self-assessment, interview, and register data) are stored on secured servers at closed networks, and access to data is logged through a unique login for an assigned list of IBBIS personnel. Physical data material (case report forms with selected interview data) is stored in locked spaces in locked facilities. Transfer of electronic data between staff members and other approved data-managing institutions is carried out using only tunnel encrypted email or encrypted USB sticks.

### Randomization

The allocation ratio between the three arms is 1:1:1. Centralized randomization will take place according to a web-based, computer-generated allocation sequence with varying block sizes kept unknown to the assessors. The Odense Patient Data Explorative Network (OPEN) is responsible for the randomization; administrative personnel in the IBBIS team perform the online randomization; and the IBBIS team leader will assign the participant to interventions and professionals.

We expect that service delivery can vary from municipality to municipality and that the process of gaining a new job from unemployment will take a longer time than returning to an existing job. Previous research has shown that diagnosis is a possible predictor of return to work [47]. Thus, the randomization is stratified according to (1) municipality, (2) employment status (on sick leave from work vs. on sick leave from unemployment), and (3) diagnosis (depression vs. anxiety).

### Blinding

Owing to the psychosocial nature of the IBBIS interventions, the participants and the professionals delivering the IBBIS interventions cannot be blinded to the group allocation. All outcomes are based on registries or self-assessed questionnaire data, and no assessor-based follow-up data will be obtained. Register data on employment status and income is created automatically through the national registries. Information on the participant’s benefit status is created through the job center management system, and benefits are granted and registered by the employment consultants in the IBBIS team.

Blinding of assessors is relevant only at the baseline interview, which takes place before group allocation. Referring personnel will likewise be blinded to the allocation sequence and block size to prevent them from anticipating the next group allocation. The researchers will be blinded to group allocation during the process of data analysis. Group allocation will be coded with names like *X*, *Y*, and *Z* to conceal the given intervention. The researcher will draw up conclusions at 6- and 12-month follow-up on the basis of six scenarios where each group (*X*, *Y*, or *Z*) has received integrated IBBIS mental health care and IBBIS vocational rehabilitation, IBBIS mental health care and standard vocational rehabilitation  and standard mental health care and vocational rehabilitation. After this, the blinding will be broken. The researcher performing analysis at 24-month follow-up will not be blind to group allocation, because any possible differences between groups will be revealed after 12-month follow-up.

### Interventions and comparisons

The IBBIS intervention team is constituted by (1) three and a half full-time care managers who are nurses, occupational therapists, physiotherapists, or social workers with mental health care experience and a minimum of 1 year of certified training in cognitive behavioral therapy (CBT), (2) three and a half full-time employment consultants who are social insurance officers from the job centers, and (3) equivalent to e.g. 0,75 psychiatrist? (alternatively, GPs or psychologists). Care managers, psychiatrists, and psychologists are employed in the mental health services, and employment consultants are employed in the job centers of the four municipalities. Care managers have a maximum momentary caseload of 25, and employment consultants have a maximum momentary caseload of 20. The IBBIS mental health care intervention is expected to have an average duration of 4 months, and the duration of the IBBIS vocational rehabilitation is expected to average 7 months.

The team delivers two separate interventions: (1) IBBIS mental health care alone and (2) IBBIS mental health care integrated with IBBIS vocational rehabilitation. Both interventions are carried out with emphasis on participant involvement through shared decision-making to improve participant satisfaction [48] and involvement of the participants’ relatives. A fidelity scale (Rie Poulsen, unpublished data) is developed and used for biannual fidelity reviews to ensure program adherence and continuous focus on program implementation and improvement. Once program fidelity is achieved, future fidelity reviews will be conducted annually.

### IBBIS mental health care and standard vocational rehabilitation

IBBIS mental health care is delivered as manualized stepped care according to national and international guidelines for treatment of depression and anxiety [19, 20, 22, 23]. The participant will be offered treatment options according to a stepped care plan, with the least invasive and least resource-demanding effective treatment offered first. One or more of the following treatment options will be provided by the care manager:

- Care plan produced in collaboration with the participant and in accordance with treatment guidelines for the specific diagnosis and relevant step
- CBT
- Regular monitoring (minimum every 2 weeks) and monthly reassessments to ensure timely changes in treatment
- Individual psychoeducation with a self-management approach
- Supplementary psychoeducational, disease-specific written material
- Involvement of relatives

Intervention modalities will be offered according to the stepped care plan (*see* Tables 2 and 3).

**Table 2:** Stepped care algorithm for depression

Diagnosis and level	Initial treatment
Mild depression	• Psychoeducation
Moderate depression	Either • Cognitive behavioral therapy (CBT) or • Pharmacological treatment and psychoeducation
Severe depression	Both • Cognitive behavioral therapy (CBT) and • Pharmacological treatment
Severe depression with complication	Referral to secondary sector

**Table 3:** Stepped care algorithm for social phobia, panic disorder, and generalized anxiety disorder

Diagnosis and level	Initial treatment
Social phobia	CBT
Panic disorder	CBT
Generalized anxiety disorder	Psychoeducation
• Without clear impact on functioning	
Generalized anxiety disorder	CBT
• With clear impact on functioning	

*CBT* Cognitive behavioral therapy

The psychiatrist of the IBBIS team is responsible for the following:

- Case supervision of care managers
- Initiation of nonmedical treatment (can be delegated to the care manager under supervision)
- Collaboration with the participant’s GP about medication and collaboration with other possible treatment providers
- Supervision in CBT if the psychiatrist has the necessary education, or else this supervision is given by, for example, a psychologist with the necessary education in CBT

Participants will receive standard vocational rehabilitation services from the local job center along with continuous control of the grounds for receiving sick leave benefit. The IBBIS team will not collaborate with job center personnel.

### Integrated IBBIS mental health care and IBBIS vocational rehabilitation

The mental health care in the integrated intervention is identical to that described in the IBBIS mental health care above. The concurrent vocational rehabilitation in IBBIS is composed of the following elements, which are delivered to meet the participant’s individual needs for vocational recovery:

- Vocational assessment of the participant’s work capacity and barriers in relation to work with a focus on readiness for return to work [49], work role functioning [50], and return to work self-efficacy [51]
- Vocational rehabilitation plan produced in collaboration with the participant and in compliance with the vocational rehabilitation manual
- Problem-solving support in returning to a current workplace and preventing recurring sick leave inspired by Dutch guidelines and the Stimulating Healthy participation and Relapse Prevention (SHARP)-at work intervention. The support is focused on a quick, stepwise return to work and problem-solving of issues related to the workplace that are barriers to return to work or impose risk factors for recurring sick leave [31, 52]
- Job search support with a focus on the best possible job match inspired by Individual Placement and Support (IPS) in accordance with the following slightly modified IPS principles:
  - Focus on competitive employment
  - Integration of mental health and employment services
  - Strong attention to participant preferences
  - Counseling about benefit programs and supported work accommodations
  - Rapid job search
  - Systematic job development
  - Time-unlimited support for work retention [53]
- Case management according to Danish sick leave benefit legislation with continuous assessment of the justification of the type and duration of sick leave benefit
- Coordination, where relevant, with other public authorities who provide social services
- Involvement of relatives

Consistency between goals in treatment and vocational rehabilitation is crucial [34, 37]. Several integrational elements ensure coherence in the participant’s process of returning to work and recovering from mental problems in the integrated IBBIS intervention:

- At least one meeting between the participant, the employment consultant, and the care manager where a joint plan for return to employment and the support from the IBBIS team is decided upon
- Collocation of all team members
- Multidisciplinary supervision of care managers and employment consultants together to enhance a continuous focus on the shared goals of the participant

The integrated services are based on the theoretical framework of relational coordination by Jody Hoffer Gittell in which timely problem-solving communication between different professionals is created by focusing on shared goals, shared knowledge, and mutual respect [54]. Unfortunately, it has not been possible to establish a shared electronic folder for IBBIS staff from different organizations to use, owing to separate, secure information technology systems, and hence written communication across sectors and municipalities can be shared only through emails.

### Training and supervision

Employment consultants and care managers all attended a 4-week training course in April 2016, comprising 1 week of joint training and 3 weeks of training in their monodisciplinary groups. Care managers are trained in all aspects of the IBBIS mental health care intervention, with a special focus on psychiatric assessments and CBT. Likewise, employment consultants are trained in all aspects of the IBBIS vocational rehabilitation intervention, with a special focus on the problem-solving method and job development. Care managers have weekly case-based supervision and CBT supervision every 2 weeks; the employment consultants have weekly supervision; and the team has monthly case-based cross-disciplinary supervision.

### Standard mental health care and standard vocational rehabilitation

Participants who will be allocated to the control group of the trial will receive standard health care by their GP and standard services in the job center. GPs can, with supervision, offer up to seven therapy sessions to patients or refer eligible patients to a private psychologist. People with depression (all age groups) and anxiety (ages 18–38) can receive payment for partial reimbursement of up to 12 sessions of individual psychological therapy. People who qualify for secondary mental health services at baseline are excluded from the IBBIS trial, but some participants will experience deteriorating symptoms during the follow-up period and eventually meet the criteria for treatment by the secondary mental health services. Mental health services offer outpatient treatment in structured treatment packages for people with depression, generalized anxiety disorder, panic disorder, and social phobia. These packages include specialized medical treatment and 6–18 sessions of therapy according to diagnosis. Therapy is most often provided in groups supplemented by individual sessions.

The job centers offer a variety of courses and support and manage the sick leave benefit case according to government legislation, which requires regular follow-up every 4 weeks, reassessment of the sick leave diagnosis after 22 weeks, self-management courses, and support to gradual return to work (in paid or unpaid jobs). Collaboration between the job center and the health care system is normally conducted through standardized sick leave certificates from the GP to the job center. Representatives from the health care system other than the individuals’ own health care providers can be used in reassessment of the individuals’ sick leave case. See Additional file 2 for an overview of the intervention-components in the three arms.

### Outcomes

The primary outcome is time from baseline to the event return to work within 12 months after baseline. Work is defined as having 4 consecutive weeks of working with a salary and with no concurrent vocational benefits. Benefit and income status is retrieved from the Danish DREAM database and the electronic income register [55]. The DREAM database is administered by Danish Agency for Labour Market and Recruitment and can be linked to a range of different registers, including the Danish income register. Returning to or achieving a flex job, a type of subsidized work, is also defined as returning to work for participants who are entitled to flex job when they enter the trial. The work-related, symptom-based, and functional secondary outcomes are presented in Table 4. All exploratory and safety measures are presented in Table 5.

**Table 4:** Primary and secondary outcomes and data collection

	Data source	Outcome	Baseline	6-Month follow-up	12-Month follow-up	24-Month follow-up
Primary	DREAM database	Time from baseline to RTW			X	
Secondary	DREAM database	Proportion in ordinary work			X	
	DREAM database	Time from baseline to RTW		X		X
	DREAM database	Time from the first day of RTW until recurrent sick leave				X
	Questionnaire	Difference in depressive symptoms measured by Beck Depression Inventory-II (BDI-II) [56]	X	X		
	Questionnaire	Difference in anxiety symptoms measured by Beck Anxiety Inventory (BAI) [59]	X	X		
	Questionnaire	Difference in stress symptoms measured by Cohen et al.’s Perceived Stress Scale (PSS) [80]	X	X		
	Questionnaire	Social and work-related function measured by WSAS [61]	X	X		

*Abbreviations: DREAM* Danish Register for Evaluation of Marginalization, *RTW* Return to work, *WSAS* Work and Social Adjustment Scale

**Table 5:** Exploratory outcomes and safety measures

Data source	Outcome	Baseline	Follow-up		
			6 Months	12 Months	24 Months
DREAM database	Weeks of work from baseline to current follow-up			X	X
Questionnaires	Symptoms of distress, anxiety, depression, and somatization measured by Four-Dimensional Symptom Questionnaire (4DSQ) [46]	X	X	X	X
	Depressive symptoms measured by Beck Depression Inventory-II (BDI-II) [56]	X		X	X
	Anxiety symptoms measured by Beck Anxiety Inventory (BAI)	X		X	X
	Stress symptoms measured by Cohen et al.’s Perceived Stress Scale (PSS) [80]	X		X	X
	Social and work-related function measured by WSAS [61]	X		X	X
	Burnout symptoms measured by Karolinska Exhaustion Scale (KES) [62]	X	X	X	X
	Health-related quality of life measured by EQ-5D-5L [81]	X	X	X	X
	General quality of life scale measured by the Flanagan QOLS [65]	X	X	X	X
	Self-efficacy concerning symptoms measured by IPQ subscale on personal control [66]	X	X	X	X
	Return to work self-efficacy measured by RTW-SE [67]	X	X	X	X
	General self-efficacy measured by General Self-Efficacy Scale (GSS) [68]	X	X	X	X
	Client satisfaction with treatment measured by CSQ-8 [69]		X		
	Presenteeism measured by Stanford Presenteeism Scale (SPS) [82]		X	X	X
	Use of therapy and therapy-like services outside IBBIS		X	X	X

*Abbreviations: DREAM* Danish Register for Evaluation of Marginalization, *WSAS* Work and Social Adjustment Scale, *EQ-5D-5L* EuroQol five-dimension five-level version, *QOLS* Quality of Life Scale, *IPQ* Illness Perception Questionnaire, *RTW-SE* Return to Work Self Efficacy scale, *CSQ* Client Satisfaction Questionnaire, *IBBIS* Integrated Mental Health Care and Vocational Rehabilitation to Individuals on Sick Leave Due to Anxiety and Depression

The Beck Depression Inventory (BDI-II) consists of 21 items to assess the intensity of depression in clinical and normal patients. Each item comprises a list of four statements (scored 0–3) arranged in increasing severity about a particular symptom of depression [56]. The Beck Anxiety Inventory (BAI) is a 21-item general questionnaire for anxiety, measuring symptoms during the last week as rated on a 4-point Likert scale from 0 to 3 [57]. The BDI-II and BAI have been shown to have excellent psychometric properties, with internal consistency around 0.9 [58, 59]. The Perceived Stress Scale (PSS) of Cohen and colleagues is a global measure of perceived stress. The scale was originally a 14-item questionnaire, and it was later modified to a 10-item questionnaire that has improved and satisfactory psychometric properties [60]. The Work and Social Adjustment Scale (WSAS) is a simple, reliable, five-item scale that measures functional impairment related to an identified problem [61], which is defined in this trial as psychological symptoms.

The 4DSQ is a 50-item questionnaire designed to assess common psychological symptoms in the last week and has a special focus on distinguishing general distress from depression, anxiety, and somatization [46]. The Karolinska Exhaustion Scale 26-item version measures the degree of exhaustion disorder and the 4 interrelated dimensions of exhaustion disorder according to the Swedish National Board of Health and Welfare: lack of recovery, cognitive exhaustion, somatic symptoms, and emotional distress [62, 63]. The EQ-5D-5L is a measure of health status in five domains: mobility, self-care, usual activities, pain/discomfort, and anxiety/depression and also includes a visual analogue scale from 0 (worst imaginable health status) to 100 (best imaginable health status) [64]. The Flanagan QOLS is a 16-item instrument that measures 5 conceptual domains of quality of life: material and physical well-being; relationships with other people; social, community, and civic activities; personal development and fulfillment; recreation; and independence [65]. The six-item personal control subscale from the revised version of the Illness Perception Questionnaire (IPQ-R) is used to evaluate the participant’s self-efficacy regarding symptom management [66]. Return to work self-efficacy (RTW-SE) is an 11-item measure for self-efficacy beliefs regarding return to work where respondents are asked to respond to statements about their jobs, imagining that they would start working tomorrow in their present emotional state [67]. The General Self-Efficacy Scale is a ten-item psychometric scale that is designed to assess optimistic self-beliefs to cope with a variety of difficult demands in life [68]. The Client Satisfaction Questionnaire (CSQ-8) is an eight-item questionnaire that is used to measure participants’ satisfaction with mental health care services and vocational rehabilitation [69]. Presenteeism refers to the state where a person attends work while sick [70] and is used as a proxy measure for returning to work while having reduced workability.

### Sample size and power calculation

The sample size of this trial is based on the primary outcome return to work rate (HR). There are, to our knowledge, no comparable Danish studies, and the sample size estimates are based on Dutch studies of comparable interventions for populations on sick leave with common mental disorders. The desired type II error risk is set at 10% (power of 90%). The mean number of days from baseline to return to work in the control group is conservatively estimated to be 210 days [71–73]. Owing to multiple testing because we will compare the three study arms, we use the Bonferroni correction for the type I error risk (α) and set it to 0.0167. An HR of 1.5 is estimated to be clinically relevant [30, 74, 75], and participants will be recruited through 639 days and followed for 365 additional days. With an allocation ratio of 1:1:1, we need 201 participants in each of the 3 arms to reject the null hypothesis that the return to work rate is equal in the control group, the IBBIS mental health care intervention, and the integrated IBBIS mental health care and IBBIS vocational rehabilitation intervention. If we fail to include 603 participants, the statistical power can be lowered to 80%, and thus only 468 participants would be needed. Power calculations (Tables 6 and 7) indicate that a sample size of 201 participants per group will be adequate to detect relevant significant differences in the secondary outcome measures with a minimum power of 80%.

**Table 6:** Power calculation for binary secondary outcomes

Outcome	Expected proportion in control group	Clinically relevant proportion in intervention group	α	Power	Test	Reference
Proportion achieving > 4 weeks of ordinary job	0.65	0.80	0.0167	0.838	χ^2^ test	[71, 83]
Proportion of > 4 weeks recurrent sick leave absence among participants who returned to work	0.19	0.08	0.0167	0.801	χ^2^ test	[84]

**Table 7:** Power calculation for linear secondary outcomes

Outcome	δ-Value for clinically relevant difference in mean	σ-Value for expected SD	α	Power	Test	Reference
Difference in depressive symptoms measured by Beck Depression Inventory (BDI)	4	11	0.0167	0.893	*t* test	[85–89]
Difference in anxiety symptoms measured by Beck Anxiety Inventory (BAI)	4	12	0.0167	0.826	*t* test	
Difference in stress symptoms measured by Cohen et al.’s Perceived Stress Scale (PSS)	5	8	0.0167	1.000	*t* test	[90–94]
Social and work-related function measured by WSAS	4	10	0.0167	0.946	*t* test	

*WSAS* Work and Social Adjustment Scale

All sample size and power calculations will be conducted using PS Power and Sample Size Calculations software [76].

### Statistical analysis plan

The primary objective of this trial is to test the null hypothesis that there is no difference in time from baseline to the event return to work between the three groups at 12-month follow-up. The alternative hypothesis is that one intervention is superior to another intervention. Because the primary outcome data are collected as register data, the data are expected to be complete. Kaplan-Meier survival curves will be computed, and the differences between the three intervention groups will be tested with Cox proportional hazards regression analysis to estimate the treatment effect as HR with 95% CI. Cox regression analysis will also be used for the secondary outcomes at 24-month follow-up: “time from return to work to recurrent sick leave” for the subpopulation of individuals who have started working and “time from baseline to return to work.”

The four continuous outcomes BDI, BAI, PSS, and WSAS are used with a repeated measures design, and the difference in the individuals’ scores between measurements will be analyzed by using linear mixed models with repeated measures and an unstructured covariance matrix if possible. All participants will be included in the analysis according to the intention-to-treat principle. Missing data from the questionnaire-based instruments will be imputed with multiple imputations if we can assume that data are missing at random or missing completely at random. The effect of missing data from all questionnaire-based outcomes will further be assessed by sensitivity analyses. All statistical tests are two-sided. All exploratory continuous outcomes will be analyzed by the same method.

A nonparametric model will be used in situations where the scores are not normally distributed. The binary outcome proportion in ordinary work will be analyzed with logistic regression. All models will be adjusted for the stratification variables. We will assess the potential interaction between time and intervention for continuous secondary outcomes. All statistical tests are two-sided.

## Discussion

This paper describes the study protocol of a randomized controlled trial comparing (1) IBBIS mental health care integrated with IBBIS vocational rehabilitation, (2) IBBIS mental health care and standard vocational rehabilitation, and (3) standard mental health care and standard vocational rehabilitation for people on sick leave because of depression or anxiety. Anxiety and depression are frequent causes of sick leave with great cost for the individual and society. This trial will test two new targeted approaches to mental health care and vocational rehabilitation and the integration of these interventions to reduce the burden of disease from anxiety and depression.

This randomized controlled trial is designed with great emphasis on minimizing bias, and reporting is done in accordance with SPIRIT guidelines [40]. This study has several methodological strengths, including that (1) the sample size is large, and hence we expect high statistical power, which allows for detection of relevant differences in both primary and secondary outcomes; (2) the randomization is done in accordance with high methodological standards; and (3) the primary outcome is based on register data, and we thus expect complete data on return to work and to avoid the common biases resulting from self-assessed data such as recall bias.

There are nonetheless some limitations. First, we expect that some contamination will occur between the IBBIS mental health care intervention and the integrated IBBIS mental health care and IBBIS vocational rehabilitation intervention, because care managers might be inclined to provide IBBIS mental health care with an undesirable emphasis on vocational recovery owing to their close collaboration with employment consultants regarding the participants in the integrated intervention. To minimize the risk of contamination for participants in the IBBIS mental health care and standard vocational rehabilitation intervention, care managers are prompted to avoid collaboration with regular job center case managers about individual cases.

Second, participants and professionals are not blinded to group allocation, and there is therefore a risk of both performance bias and subject expectancy bias. These likely biases are difficult to prevent and will be included in the interpretation of the results.

Third, implementation of structured interventions in multicenter designs has previously been shown to be difficult. Several context factors affect the implementation of the intervention [77], and some variation in the delivered services between the Danish municipalities is expected [78]. We are attempting to minimize the bias from the possibly skewed implementation by stratifying the randomization for municipality. To address the possible differences in effects between municipalities, we will also conduct fidelity reviews to explicate differences in implementation.

Fourth, multidisciplinary teams have previously been shown to be difficult to establish [78], and the collaboration in integrated care can be difficult to implement because it has to be established and maintained on macro-, meso-, and microlevels in all municipalities [79]. Thus, we expect the teams to perform better at the end of the trial period than at the beginning, which can explain a missing or minimal effect from the interventions. This will be examined by analyzing the possible interaction between intervention and time.

Fifth, a methodological limitation is the fact that we cannot measure the effect of the IBBIS vocational rehabilitation alone. A 2 × 2 factor design is unfortunately not suitable when the intervention components are expected to interact in synergy in the integrated intervention.

Sixth, standard mental health care and standard vocation rehabilitation for people with stress-related disorders are very scarcely described in Denmark. Thus, a limitation in the study design is the limited knowledge about the quality and quantity of the control intervention. To improve the possibilities for comparison between the three interventions, three questions about the participants’ use of therapy and therapy-like services outside IBBIS have been added to the self-assessment scheme.

Seventh, the IBBIS professionals are attached to different organizations and are thus subject to different legislative regimes, and journaling must be conducted in different systems. IBBIS team members’ only means of sharing written communication is therefore through emails, which can be seen as a barrier for cross-disciplinary communication and thus a limitation in the implementation of integrated services. Co-location of IBBIS team members is nonetheless emphasized in the IBBIS model to promote frequent and problem-solving face-to-face communication between professionals and to enhance shared goals, shared knowledge, and mutual respect.

If this trial shows that the IBBIS mental health care intervention is superior to standard treatment, these positive results will support the further development of enhanced community-based mental health care for people on sick leave, and a wider implementation of treatment teams similar to IBBIS can be recommended. If this trial shows that integrated IBBIS mental health care and IBBIS vocational rehabilitation is superior to standard treatment or IBBIS mental health care alone, the positive results will support the assumption that integrated care is not only a perceived need from the target group but also an effective way of supporting people in their vocational recovery. If any of the IBBIS interventions prove to be superior to standard services, the findings can urge policy makers in similar contexts to collaborate on seeking solutions across sectors when the economic benefits from improved return to work accrue to the social/vocational sector or the employer, whereas the costs of improving access to therapy are placed within the health care sector. If the standard intervention is superior regarding return to work, we have a further incentive to attempt to improve treatment and vocational care. It can be considered if return to work rates have reached a maximum.

This study can contribute new knowledge on integrated vocational and health care interventions in welfare societies with separate health care and occupational sectors, as well as prevention of recurrent sickness absence among people with depression and anxiety in general. If any of the IBBIS interventions prove to be superior to standard services, the findings can urge policy makers in similar contexts to collaborate on seeking solutions across sectors when the economic benefits from improved return to work accrue to the social/vocational sector or the employer, whereas the costs of improving access to therapy are placed within the health care sector.

### Trial status

The IBBIS depression and anxiety trial was initiated in April 2016, and as of 7 November 2017, 395 participants had been recruited. This protocol is version 2.0. Trial recruitment is expected to end on 31 December 2017.

## Additional files

Additional file 1: Full Standard Protocol Items: Recommendations for Interventional Trials (SPIRIT) figure. (JPG 70 kb)

Additional file 2: SPIRIT 2013 checklist: recommended items to address in a clinical trial protocol and related documents. (DOC 121 kb)

## Abbreviations

4DSQ: Four-Dimensional Symptom Questionnaire
AWaC: At Work and Coping
BAI: Beck Anxiety Inventory
BDI-II: Beck Depression Inventory II
CBT: Cognitive behavioral therapy
CONSORT: Consolidated Standards of Reporting Trials
CRF: Case report form
CSQ-8: Client Satisfaction Questionnaire
DREAM: Danish Register for Evaluation of Marginalization
EQ-5D-5L: EuroQol five-dimension five-level version
GP: General practitioner
GSS: General Self-Efficacy Scale
IBBIS: Integrated Mental Health Care and Vocational Rehabilitation to Individuals on Sick Leave Due to Anxiety and Depression
IPQ-R: Illness Perception Questionnaire–Revised
IPS: Individual Placement and Support
KES: Karolinska Exhaustion Scale
M.I.N.I.: Mini International Neuropsychiatric Interview
MMSE: Mini Mental State Examination
OPEN: Odense Patient data Explorative Network
PSS: Perceived Stress Scale
QOLS: Quality of Life Scale
RTW-SE: Return to Work Self-Efficacy scale
SPIRIT: Standard Protocol Items: Recommendations for Interventional Trials
SPS: Stanford Presenteeism Scale
WSAS: Work and Social Adjustment Scale
