# Supplementary figures and images for: Integrated mental health care and vocational rehabilitation to improve return to work rates for people on sick leave because of depression and anxiety (the Danish IBBIS trial): study protocol for a randomized controlled trial
Source: Trials. 2017 Dec 2;18:578. doi: 10.1186/s13063-017-2272-1 (PMC5712198; doi:10.1186/s13063-017-2272-1)

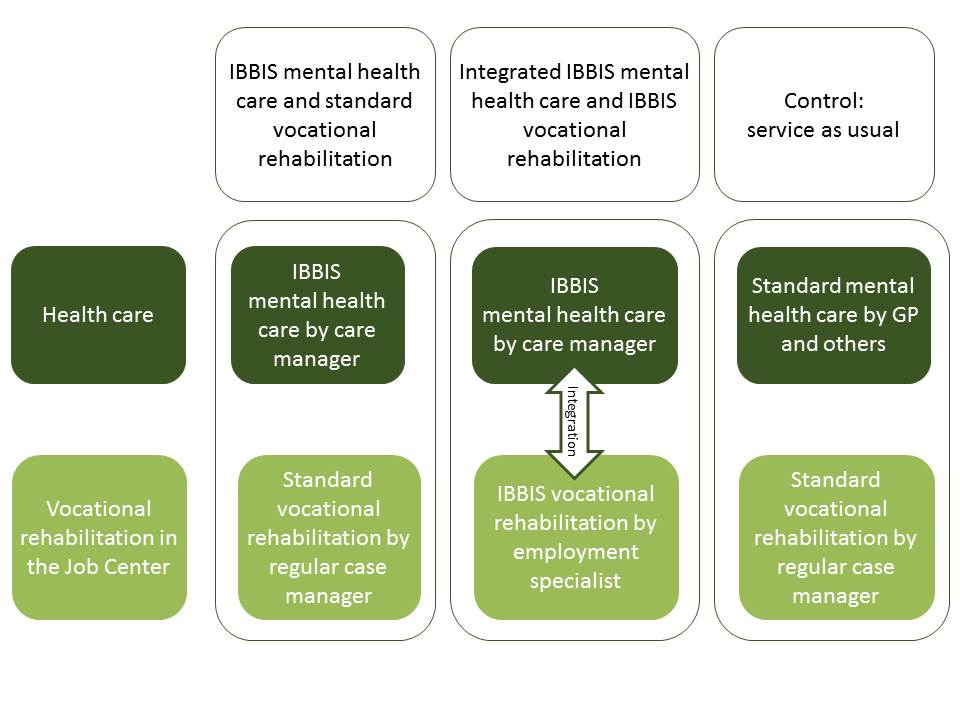

Supplement: Supplementary file 1 — Full Standard Protocol Items: Recommendations for Interventional Trials (SPIRIT) figure. (JPG 70 kb) [file 13063_2017_2272_MOESM1_ESM.jpg]
